# Dexmedetomidine Alleviates Intracerebral Hemorrhage-Induced Anxiety-Like Behaviors in Mice Through the Inhibition of TRPV4 Opening

**DOI:** 10.3389/fphar.2022.852401

**Published:** 2022-04-01

**Authors:** Ping An, Xiao-Chun Zhao, Man-Jia Liu, Yu-Qing You, Jing-Ya Li, He-Song Gong

**Affiliations:** ^1^ Department of Neurobiology, School of Life Science, China Medical University, Shenyang, China; ^2^ Department of Anesthesiology, School and Hospital of Stomatology, China Medical University, Shenyang, China; ^3^ Department of Anesthesiology, ShengJing Hospital of China Medical University, Shenyang, China

**Keywords:** dexmedetomidine, intracerebral hemorrhage, TRPV4, astrocyte, anxiety

## Abstract

Post-stroke anxiety severely affects recovery in patients with intracerebral hemorrhage (ICH). Dexmedetomidine (Dex), a highly selective alpha 2 adrenal receptor (α2-AR) agonist, was recently found to exert an excellent protective effect against mental disorders including anxiety. The transient receptor potential vanilloid 4 (TRPV4) channel is involved in a series of diseases such as asthma, cancer, anxiety, and cardiac hypertrophy. This study examines whether Dex improved ICH-induced anxiety via the inhibition of TRPV4 channel opening. A rodent model of moderate ICH in the basal ganglia was established using autologous blood injection (20 μl). Mice were treated with Dex (25 μg/kg, intraperitoneal injection) every day for 3 days post-ICH. GSK1016790A (1 μmol/2 μl), an agonist of TRPV4, was administered *via* the left lateral ventricle. Thirty days post-ICH, post-stroke anxiety was evaluated by elevated plus-maze and open-field tests. Following behavioral tests, superoxide dismutase (SOD), malondialdehyde (MDA), astrocytic activation, and A1-and A2-type astrocytes were determined. Primary astrocytes were exposed to hemin to simulate ICH *in vitro*. Compared with sham-treated mice, Dex administration ameliorates ICH-induced decreases of distance and time in the open-arm, reduces distance and time in the central zone, increases astrocytic activation and A1-type astrocytes, elevates MDA content, downregulates total SOD contents, and decreases A2-type astrocytes. However, GSK1016790A partially reversed the neuroprotective effects of Dex. In addition, Dex significantly inhibited hemin-induced astrocytic activation *in vitro*. Dex improves ICH-induced anxiety-like behaviors in mice, and the mechanism might be associated with the inhibition of TRPV4-channel opening.

## Introduction

Although spontaneous intracerebral hemorrhage (ICH) represents a relatively small percentage of all types of strokes, the resultant high mortality and disability rates impose a heavy economic and social burden (Murayama et al., 2016). Ischemic injury induced by hematoma mechanical compression, blood–brain barrier disruption that results from hemoglobin, and metabolism disorders caused by inflammatory response have been suggested to lead to secondary brain damage (Wang et al., 2018; Zhu et al., 2019). Mental disorders following a moderate ICH such as anxiety, have recently received attention because of secondary brain injury (Koivunen et al., 2015). Numerous publications have demonstrated that post-stroke anxiety severely hinders patients with an ICH from returning to society (Sallinen et al., 2019). The potential functions of anti-inflammatory agents including cell-permeable peptides, and anti-oxidative stress agents including hydrogen gas have been widely demonstrated in animal models of ICH (Luo et al., 2019; An et al., 2022). Effective strategies or drugs for the treatment of ICH are not yet available in clinical practice.

Dexmedetomidine (Dex) induces norepinephrine releases on the presynaptic membrane via activating alpha 2 adrenal receptor, which exerts analgesic, sedative, sympatholytic, and anxiolytic effects (Keating, 2015). Recently, Dex has been reported to inhibit the production of inflammatory cytokines in multiple organs including the brain, kidney, and lungs (Zhang Y. et al., 2018; Bao and Dai, 2020; Mei et al., 2021). In addition, Chen et al. (2018) showed that the protective effects of Dex are associated with the attenuation of apoptosis and the inhibition of oxidative stress. It is worth noting that Dex significantly suppresses inflammasome activation and maintains the integrity and permeability of the blood–brain barrier under the ICH condition (Song and Zhang, 2019). Interestingly, Dex can improve a patient’s anxiety, and reduce the incidence rate of cognitive dysfunction (Ji et al., 2014; Wu and Kang, 2020). However, little is known about the potential anxiolytic effects of Dex in mild ICH, including its potential involvement in antioxidative and anti-inflammatory responses.

Alpha 2 adrenal receptor is co-localized with transient receptor potential vanilloid (TRPV) receptor, and it was suggested that dexmedetomidine contributes to the anti-nociceptive action via inhibition of TRPV (Lee et al., 2020). As a member of the transient receptor potential (TRP) superfamily, it has been demonstrated that the TRPV4 channel exists widely in the central nervous system (Kitchen et al., 2020). A variety of stimuli including moderate temperature, mechanical stimulation, and cell swelling can activate the opening of TRPV4 channels, subsequently inducing the influx of the calcium ion (Vriens et al., 2004). Changes in the calcium ion concentration further regulate the integrity of the blood-cerebrospinal fluid barrier, cell volume, tension of cerebral vessels, and excitability of neurons (Dunn et al., 2013). Under brain ischemia and reperfusion conditions, it was reported that neuronal death and brain edema are associated with the participation of TRPV4 channels. Moreover, the inhibition of the TRPV4-channel opening also exhibits a neuroprotective effect in ICH (Shen et al., 2019). Astrocyte, one of the most widely distributed cell types in the brain, has been suggested to involve in structural and functional modifications of neurons (Liu and Chopp, 2016). Astrocyte inflammation and dysfunction are related to the pathogenesis of anxiety-associated diseases (Yang et al., 2016; Martin-Fernandez et al., 2017). Intriguingly, TRPV4 channels also plays a vital role in the regulation of astrocytes in a series of diseases such as hypertension and stroke (Filosa et al., 2013; Wang et al., 2019). Based on these findings, we examined whether TRPV4 channels in astrocytes participate in the process of ICH-induced pathophysiology.

Therefore, we hypothesized that Dex administration ameliorated anxiety-like behaviors in mice with ICH. In addition, we also investigated whether TRPV4 channels in astrocytes were involved in the neuroprotective effects of Dex.

## Materials and Methods

### Experimental Animals

Adult male C57BL/6 mice (25–30 g, 10–12 weeks) were purchased from Changsheng Biotechnology Co., Ltd (Benxi, Liaoning, China). All mice were allowed to take food and water freely, and kept in a 12-h alternating light and dark facility at 25 ± 1°C and a humidity of 50–70%. The tests involving animals were performed according to the guidelines of the Animal Ethics Committee of China Medical University.

### Grouping and Treatments

**FIGURE 1:**
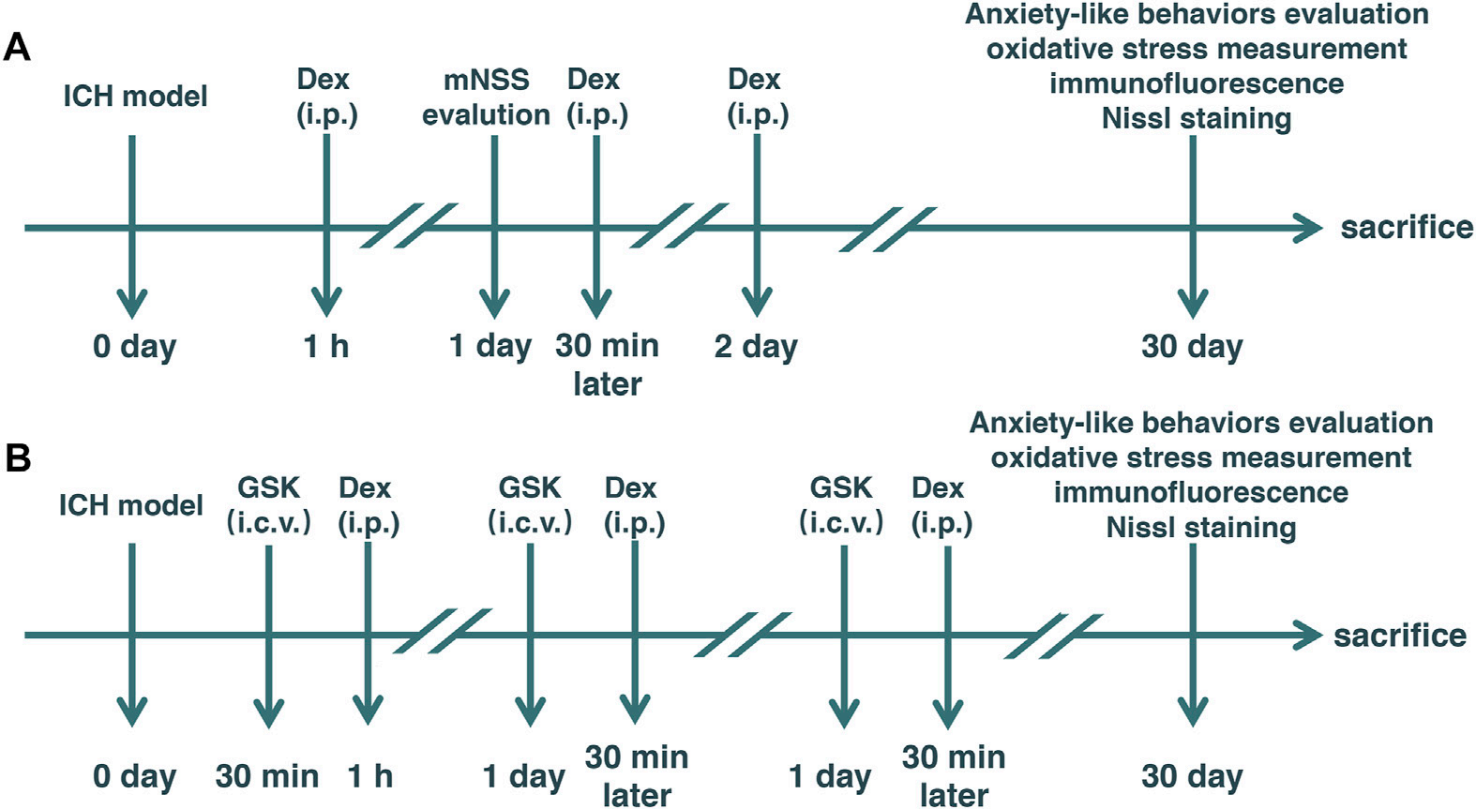
Experimental schematic diagram. Mice with intracerebral hemorrhage (ICH) and dexmedetomidine (Dex) administration. Elevated plus-maze and open-field tests were used to evaluate anxiety-like behaviors. ICH: Rats received an injection of autologous arterial blood (20 μl) in the basal ganglia. mNSS: modified neurological severity score **(A)** Dex: 25 μg/kg Dex using intraperitoneal injection was administered post-ICH daily for 3 days. **(B)** GSK: GSK1016790A, an agonist of transient receptor potential vanilloid 4 (TRPV4), injected into the left lateral ventricle 30 min before Dex administration. Sham: Mice underwent identical surgery except for ICH treatment.

Thirty-six adult male C57BL/6 mice were divided into six groups according to a computer-based randomization in experiment 1 (*n* = 12) as follows: 1) sham + vehicle, 2) ICH + vehicle, and 3) ICH + Dex. Autologous blood injection in the basal ganglia was used to establish a mild ICH model in mice. Based on previous studies (Gu et al., 2011; Liang et al., 2017) and our preliminary experiments, 25 μg/kg of Dex (Jiangsu Hengrui Medicine Co., Ltd., Jiangsu, China) was administered using intraperitoneal injection post-ICH daily for 3 days. Mice in sham + vehicle and ICH + vehicle groups received an equal volume of saline *via* intraperitoneal injection (Figure 1A).

Twenty-four adult male C57BL/6 mice were divided into two groups according to a computer-based randomization in experiment 2 (*n* = 12) as follows: 1) ICH + Dex + GSK and 2) ICH + Dex + vehicle. GSK1016790A (GSK, 1 μmol/2 μl, HY-19608, MedChemExpress, United States), an agonist of TRPV4, was dissolved in 10% dimethyl sulfoxide (DMSO) (D8418, Beyotime, China) and 90% corn oil (HY-Y1888, MedChemExpress, United States), and injected into the left lateral ventricle (stereotaxic coordinates: 1.0 mm lateral to the midline, 0.3 mm posterior to the bregma, and 2.5 mm below the skull) 30 min before Dex administration. Vehicle was administered with an equivalent volume of DMSO plus corn oil as a control (Figure 1B).

### Intracerebral Hemorrhage Model

Following anesthetic induction with 7–8% sevoflurane, mice were fixed on a warming pad (temperature, 38 ± 1°C) with 2–3% sevoflurane. After sterilization, shaving, and exposing the skull, we made a hole on the skull (1 mm in diameter and 2 mm to the right of the bregma). We infused 5 μl of autologous arterial blood obtained from the central tail artery using a microsyringe into the basal ganglia (0.4 mm anterior and 2.0 mm right lateral to the bregma) at 2.8 mm ventral to the skull. Five minutes later, the needle was further deepened to 3.5 mm ventral to the skull, and 15 μl of autologous arterial blood was infused. Five minutes later, the needle was slowly withdrawn. Bone wax was used to cover the drill site, and then the skin was closed through the interrupted suture. In mice with sham treatment, the sham group, 20 μl of sterile saline (0.9%) was administered into the basal ganglia.

### Neurological Behavioral Assessment

The modified neurological severity score (mNSS) was used to evaluate the severity of ICH at 24 h and 30 d after surgical exposure. Four functions including sensory (visual, tactile, and proprioceptive tests), motor (test by raising mouse by the tail and placing it on the floor), balance (beam-balance tests), and reflex (reflexes absent and abnormal movements) were performed by two observers who were blinded to the experiments on a scale of 0–18. Severe, moderate, and mild ICH were classified based on scores of 1–6, 7–12, and 13–18, respectively.

### Open Field Test

At 30 d after ICH, the open-field test (OFT) was used to assess the anxiety-like behaviors in mice. After acclimatizing to the room for 1 h, mice were placed into an acrylic container (length: 60 cm; width: 60 cm; height: 60 cm). At the beginning of each trial, each mouse was placed at the bottom right of the container. After 1 min of adaptation, the behaviors of mice were recorded for 5 min. The movement was tracked by a video camera fixed 1 m above the container. The apparatus was cleaned with 75% alcohol to remove the smell. The distance, speed, time, and distance in the central zone were recorded and further analyzed.

### Elevated Plus Maze Test

After the OFT was completed, the elevated plus-maze test (EPMT) was also used to measure the anxiety-like behaviors in mice. The elevated plus-maze was constituted by two closed arms (with walls of 40.5 cm in height) and two open arms (without walls). The apparatus was placed on a position that was elevated 70 cm from the ground. Mice facing the open arm were positioned in the center of the apparatus, and their behaviors were recorded for 5 min. The apparatus was cleaned with 75% alcohol to remove the smell. The distance and time spent in the open arm were used to analyze for anxiety-like behaviors.

### Culture of Primary Astrocytes and Treatment Administration

We obtained primary mouse astrocytes from newborns within 24 h. Cortical tissues were minced, digested with 0.025% pancreatic enzymes, and filtered using 200 mesh. Following centrifugation, we resuspended precipitates in Dulbecco’s modified eagle medium (DMEM) medium with 10% fetal bovine serum (FBS). Primary cell cultures were incubated in a humidified atmosphere of 95% air and 5% CO_2_ at 37°C. On day *in vitro* (DIV) 3, half of the culture medium was removed and replaced with fresh DMEM medium with 10% FBS. On DIV 7, astrocyte cultures were purified by shaking with 270 r/min for 2 h at 37°C in an orbital shaker. Cells in the culture were shown to be astrocytes with a purity of 97 ± 2% after characterization by immunostaining using a primary anti-autoimmune glial fibrillary acidic protein (GFAP) antibody. On DIV 9, the astrocytes were randomly divided into five groups: 1) control + vehicle 1; 2) hemin + vehicle 1; 3) hemin + Dex; 4) hemin + Dex + vehicle 2; 5) hemin + Dex + GSK. 30 μM of hemin (HY-19424, MedChemExpress, United States) dissolved in DMSO for 12 h was used to imitate ICH *in vitro*. Dex (1 μM) and GSK1016790A (1 μM) were administered into cell culture 30 min after hemin treatment. 0.9% saline and DMSO were used as vehicle 1 and vehicle 2, respectively.

### Cell Viability Assay and Lactate Dehydrogenase Assay

Cell counting kit-8 (CCK-8) (C0038, Beyotime, China) was used to measure cell viability. In addition, a lactate dehydrogenase (LDH) cytotoxicity assay kit (C0016, Beyotime, China) was used to evaluate cell injury. Astrocytes plated into a 96-well plate were treated with the above-mentioned exposures. Then the experiment continued according to the instructions for the usage of kits. Absorbances at 450 and 490 nm were read using a microplate absorbance reader (DeTie, Nanjing, China). Acquired data were expressed as the percentage of the control + vehicle 1 group.

### Measurements of Reactive Oxygen Species and Superoxide Dismutase Contents

Contents of malondialdehyde (MDA) and superoxide dismutase (SOD) were measured using MDA (thiobarbituric acid method) (A003, Jiancheng, Nanjing, China) and SOD (WST-1 method) (A001, Jiancheng, Nanjing, China) assay kits according to the manufacturers’ instructions, respectively.

### Immunofluorescence

Once behavior tests ended, mice were anesthetized with sevoflurane, and perfused with 4°C saline containing heparin and 4% paraformaldehyde in sequence. The cerebral tissue was rapidly removed, dehydrated, and embedded in the paraffin. Then, 4-μm thick coronal paraffin slices were used for immunofluorescence assay. Primary antibodies including mouse monoclonal anti-GFAP (catalog #AF0156, lot #061220201221, 1:100; Beyotime, Shanghai, China), rabbit polyclonal anti-C3 complement (1:100; Beyotime, Shanghai, China), and rabbit polyclonal anti-S100β (1:100; Beyotime, Shanghai, China) were assayed for 12 h at 4°C. Cy3-conjugated goat anti-mouse IgG (1:500; Beyotime, Shanghai, China) and fluorescein isothiocyanate-conjugated goat anti-rabbit IgG (1:500; Beyotime, Shanghai, China) were used as secondary antibodies. Nuclei were labeled with 4′,6-diamidino-2-phenylindole (DAPI) (10 μg/ml, C1002, Beyotime, China) after the last wash with phosphate-buffered saline. Fluorescent signals were detected with a fluorescence microscope (DP70, Olympus, Japan). Fluorescence analysis and cell count were performed using ImageJ software. Six fields with a magnification of 200× in three sections were randomly selected from each group. Sholl analysis was performed to assess branch tips in a given astrocyte indicated by GFAP-positive cells. The percentage of GFAP- and C3 complement-positive cells, and GFAP- and S100β-positive cells were analyzed using Image-Pro Plus 6.0 (NIH, Bethesda, MD, United States).

### Statistical Analysis

All data are presented as a mean ± standard deviation (SD), then analyzed using GraphPad prism 8.0 software (GraphPad Software Inc., San Diego, CA, United States). The homogeneity of variance hypothesis was identified using the Levene test. Statistical comparisons were made using one-way ANOVA following Tukey *post hoc* test. For all comparisons, statistical significance was set at a *p* < 0.05.

## Results

### Dex Administration Attenuated Anxiety-Like Behaviors After ICH

**FIGURE 2:**
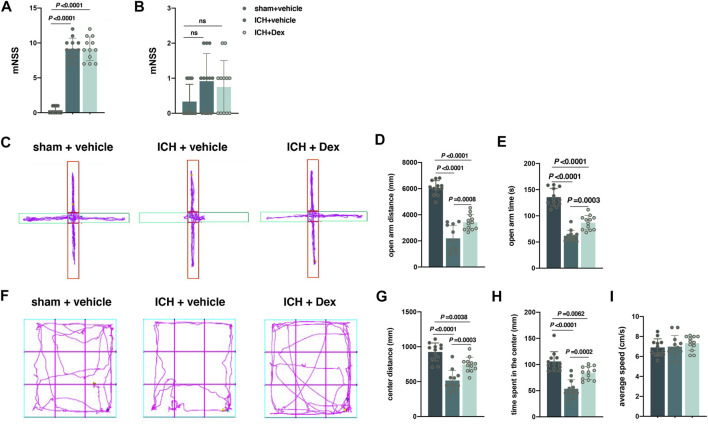
Dexmedetomidine administration attenuated anxiety-like behaviors after ICH exposure. **(A,B)** Changes in the mNSS caused by the indicated stimuli 1 and 30 d post-ICH. **(C)** Computer printouts of shift trajectories in the elevated plus-maze test caused by the indicated stimuli 30 d post-ICH. **(D,E)** Distance and time in the open-arm during the elevated plus-maze test results caused by the indicated stimuli 30 d post-ICH. **(F)** Computer printouts of shift trajectories in the open-field caused by the indicated stimuli 30 d post-ICH. **(G–I)** Distance and time in the central zone, and average speed during the open-field test results caused by the indicated stimuli 30 d post-ICH. Data are presented as the mean ± SD (*n* = 12). Sham, ICH, and Dex are described above.

Evaluation of behavioral performance was carried out at 24 h and 30 d after ICH. It was shown that mNSS at 24 h after ICH in the sham + vehicle group was significantly lower than that in the ICH + vehicle and ICH + Dex groups (sham + vehicle *versus* ICH + vehicle, *p* < 0.0001; sham + vehicle *versus* ICH + Dex, *p* < 0.0001; Figure 2A). However, there was no remarkable difference in the mNSS between ICH + vehicle and ICH + Dex groups (Figure 2A). In addition, there was also no difference in the mNSS between the three groups (Figure 2B).

Anxiety, a negative emotional state, has been reported to be related with the perception of potential or ambiguous threat (Rombolà et al., 2017). The distance and time in the open arm indicated by the EPMT and the distance and time in the central zone indicated by the OFT were used to evaluate the negative emotional state mentioned above. Compared with sham-treated mice, ICH-treated mice exhibited a significant attenuation of distance (*versus* sham + vehicle, *p* < 0.0001; Figures 2C,D) and time (*versus* sham + vehicle, *p* < 0.0001; Figures 2C,E) in the open-arm time. In the OFT, mice exposed to ICH walked less distance (*versus* sham + vehicle, *p* < 0.0001; Figures 2F,G) and spent less time (*versus* sham + vehicle, *p* < 0.0001; Figures 2F,H) in the central zone than that of the sham animals in a 5-min test session. However, compared with ICH-treated animals, Dex administration attenuated the open-arm distance (*versus* ICH + vehicle, *p* = 0.0008; Figures 2C,D) and time (*versus* ICH + vehicle, *p* = 0.0003; Figures 2C,E) in the EPMT, as well as the distance to the center (*versus* ICH + vehicle, *p* = 0.0003; Figures 2F,G) and time (*versus* ICH + vehicle, *p* = 0.0002; Figures 2F,H) in the OFT. In addition, there was no significant difference of average speed in the EPMT and OFT between the above three groups (Figures 2F,I).

### Dex Administration Mitigated Oxidative Stress and Neuronal Injury Post-ICH

**FIGURE 3:**
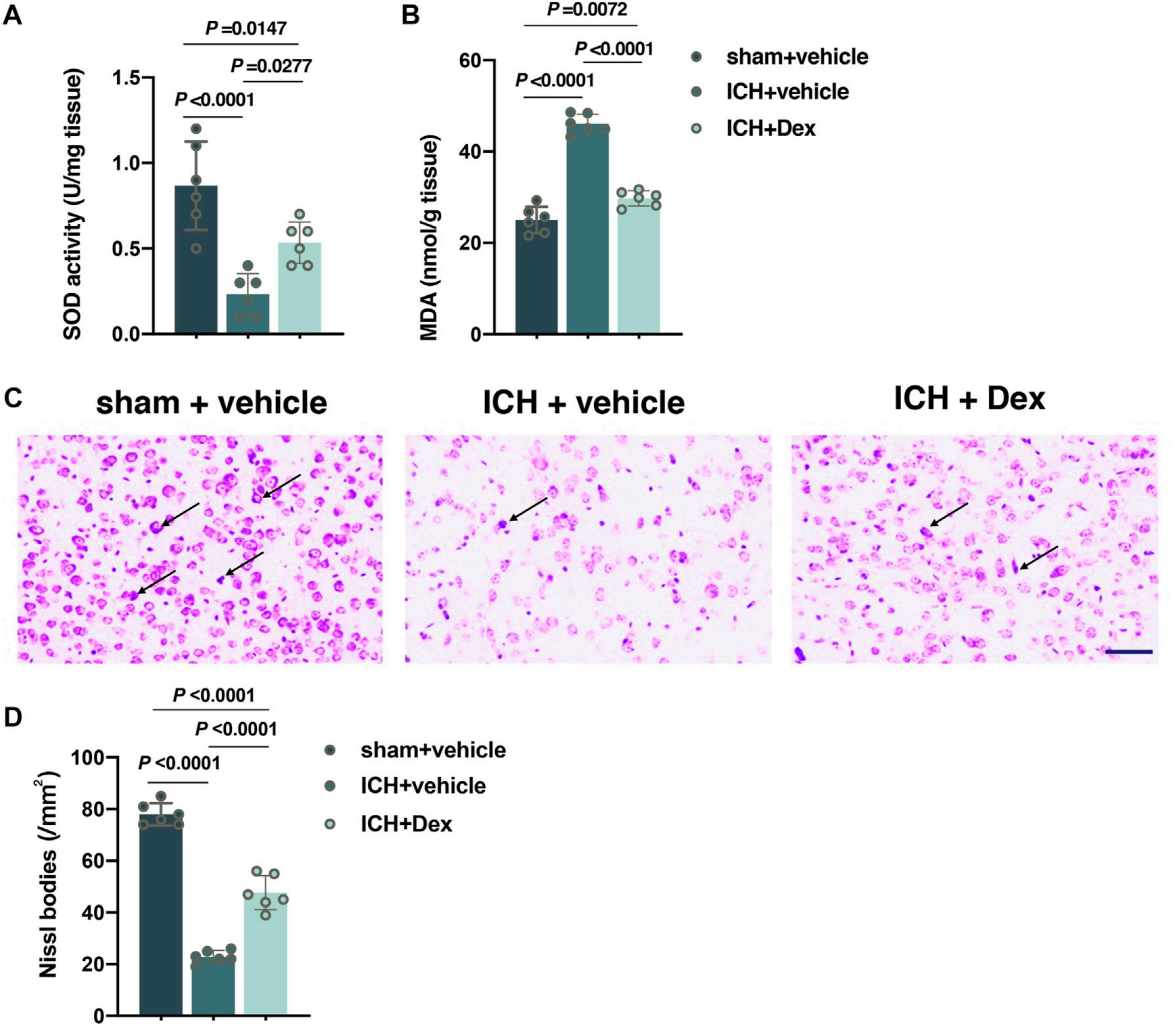
Dexmedetomidine administration attenuated oxidative stress and neuronal injury after ICH exposure. **(A,B)** Changes in the total SOD and MDA contents around the hematoma in the corpus striatum caused by the indicated stimuli. **(C,D)** Representative photomicrographs of Nissl staining and Nissl bodies around the hematoma in the corpus striatum caused by the indicated stimuli. Arrows indicate Nissl bodies. Data are presented as the mean ± SD (*n* = 6). Sham, ICH, and Dex are described above.

The total SOD and MDA contents around the hematoma in the corpus striatum were used to assess oxidative stress at 30 days after ICH. As shown in Figure 3, compared to mice exposed to sham treatment, ICH resulted in a significant reduction in the total SOD content (*versus* sham + vehicle, *p* < 0.0001; Figure 3A), but an increase in the MDA content (*versus* sham + vehicle, *p* < 0.0001; Figure 3B) occurred in mice treated with ICH plus vehicle. However, compared to mice with ICH exposure, the content of total SOD was partially restored (*versus* ICH + vehicle, *p* = 0.0277; Figure 3A), but the content of MDA (*versus* ICH + vehicle, *p* < 0.0001; Figure 3B) was heavily attenuated in mice with ICH plus Dex treatment. As a neural characteristic structure, the number of Nissl bodies reflects the state of neurons. Compared with sham animals, ICH exposure remarkably decreased the number of Nissl bodies around the hematoma in the corpus striatum (*versus* sham + vehicle, *p* < 0.0001; Figures 3C,D) at 30 days after ICH, while Dex treatment partially reversed these changes (*versus* ICH + vehicle, *p* < 0.0001; Figures 3C,D).

### Dex Administration Mitigated the Activation of Astrocytes Post-ICH

**FIGURE 4:**
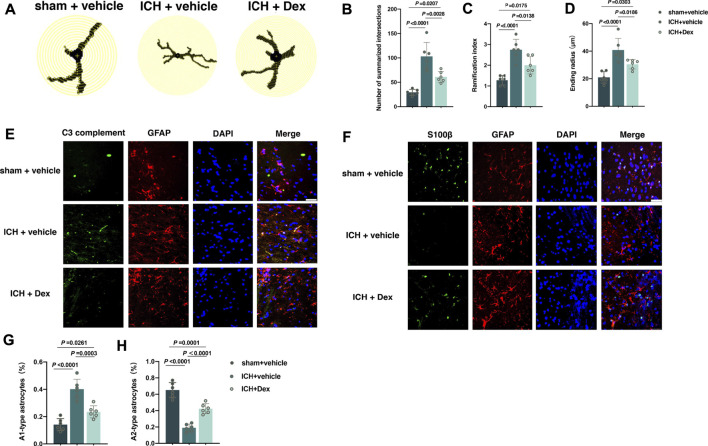
Dexmedetomidine administration attenuated the activation of astrocytes after ICH exposure. **(A)** Representative detailed Sholl analysis of individual reactive astrocytes around the hematoma in the corpus striatum caused by the indicated stimuli. The interval of the concentric circles is 1 μm. **(B–D)** Changes in the sum of intersects, ramification index, and ending radius in the corpus striatum caused by the indicated stimuli. **(E)** Representative photomicrographs of GFAP and C3 complement plus DAPI staining (GFAP, red; C3 complement, green; DAPI, blue). 200× magnification, scale bar = 50 μm. **(F)** The percentage of GFAP combined with C3 complement-positive cells (A1-type) caused by the indicated stimuli. **(G)** Representative photomicrographs of GFAP and S100β plus DAPI staining (GFAP, red; S100β, green; DAPI, blue). 400× magnification, scale bar = 50 μm. **(H)** The percentage of GFAP combined with S100β-positive cells (A2-type) caused by the indicated stimuli. Data are presented as the mean ± SD (*n* = 6). Sham, ICH, and Dex are described above.

The activation of astrocytes around the hematoma in the corpus striatum post-ICH was measured using detailed Sholl analysis of individual reactive astrocytes and the transformation from A2-to A1-type astrocytes. From the results of immunofluorescence, it was shown that the sum of intersects (*p* < 0.0001; Figures 4A,B), ramification index (*p* < 0.0001; Figures 4A,C), and ending radius (*p* < 0.0001; Figures 4A,D) significantly elevated in mice treated with ICH + vehicle compared to sham + vehicle (*versus* sham + vehicle), whereas Dex administration partially reversed the increase in the sum of intersects (*p* = 0.0028; Figures 4A,B), ramification index (*p* = 0.0138; Figures 4A,C), and ending radius (*p* = 0.0186; Figures 4A,D) (*versus* ICH + vehicle). Recent evidence have demonstrated that there are two different types of reactive astrocytes including the C3 immunopositive neurotoxic A1-type astrocytes and the S100β immunopositive neuroprotective A2-type astrocytes (King et al., 2020; Kaur and Conti, 2021). We found that A1-type astrocytes indicated by C3 complement combined with GFAP were heavily increased in mice exposed to ICH compared to sham (*versus* sham + vehicle, *p* < 0.0001; Figures 4E,G). However, Dex administration significantly reduced A1-type astrocytes in mice exposed to ICH plus Dex (*versus* ICH + vehicle, *p* = 0.0003; Figures 4E,G). In addition, A2-type astrocytes indicated by S100β combined with GFAP were remarkably reduced in mice exposed to ICH compared with sham (*versus* sham + vehicle, *p* < 0.0001; Figures 4F,H), while Dex administration partially restored the number of A2-type astrocytes (*versus* ICH + vehicle, *p* < 0.0001; Figures 4F,H).

### TRPV4 Signal Responsible for Dex-Mediated Neuroprotective Effects Post-ICH

**FIGURE 5:**
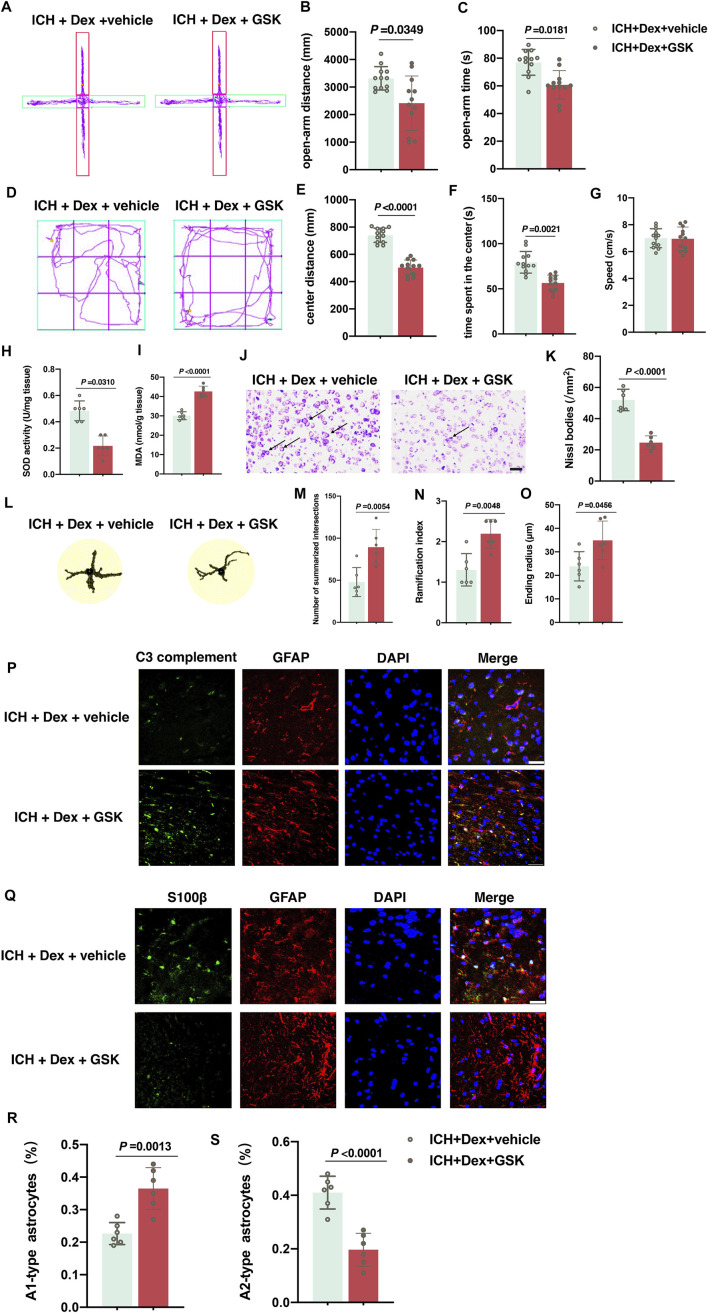
GSK1016790A reversed the neuroprotective effects induced by dexmedetomidine-post-ICH. **(A)** Computer printouts of shift trajectories in the elevated plus-maze caused by the indicated stimuli 30 d post-ICH. **(B,C)** Distance and time in the open-arm during the elevated plus-maze results caused by the indicated stimuli 30 d post-ICH. **(D)** Computer printouts of shift trajectories in the open-field caused by the indicated stimuli 30 d post-ICH. **(E–G)** Distance and time in the central zone, and average speed during the open-field test results caused by the indicated stimuli 30 d post-ICH. **(H,I)** Changes in the total SOD and MDA contents around the hematoma in the corpus striatum caused by the indicated stimuli. **(J,K)** Representative photomicrographs of Nissl staining and Nissl bodies around the hematoma in the corpus striatum caused by the indicated stimuli. Arrows indicate Nissl bodies. **(L)** Representative detailed Sholl analysis of individual reactive astrocytes around the hematoma in the corpus striatum caused by the indicated stimuli. **(M–O)** Changes in the sum of intersects, ramification index, and ending radius in the corpus striatum caused by the indicated stimuli. **(P)** Representative photomicrographs of GFAP and C3 complement plus DAPI staining (GFAP, red; C3 complement, green; DAPI, blue). 400× magnification, scale bar = 50 μm. **(Q)** Representative photomicrographs of GFAP and S100β plus DAPI staining (GFAP, red; S100β, green; DAPI, blue). 200× magnification, scale bar = 50 μm. **(R)** The percentage of GFAP combined with C3 complement-positive cells (A1-type) caused by the indicated stimuli. **(S)** The percentage of GFAP combined with S100β-positive cells (A2-type) caused by the indicated stimuli. Data are presented as the mean ± SD (*n* = 12 or 6). ICH, Dex, and GSK are described above.

Subsequently, the TRPV4 signal was determined to play a vital role in the neuroprotective effects induced by the Dex administration under the ICH condition. Our data showed that the opening of TRPV4 channels activated by GSK1016790A heavily abolished the beneficial effects of Dex administration in attenuating anxiety-like behaviors. All the data were evaluated by ANOVA after merging with sham, ICH + vehicle and ICH + Dex groups. In the EPMT, it was shown that GSK1016790A significantly reduced the distance (*p* = 0.0349; Figures 5A,B) and time (*p* = 0.0181; Figures 5A,C) in the open-arm in mice treated with ICH plus Dex and GSK1016790A exposure compared to ICH plus Dex and vehicle (*versus* ICH + Dex + vehicle). Similarly, OFT results also indicated that GSK1016790A heavily attenuated the distance (*p* < 0.0001; Figures 5D,E) and time (*p* = 0.0021; Figures 5D,F) in the central zone (*versus* ICH + Dex + vehicle). Intriguingly, there was an obvious reduction of the total SOD content (*p* = 0.0310; Figure 5H), but an elevation of the MDA content (*p* < 0.0001; Figure 5I) and attenuation of Nissl bodies (*p* < 0.0001; Figures 5J,K), which reflect aggravated oxidative stress and neuronal injury (*versus* ICH + Dex + vehicle). In addition, we found that GSK1016790A treatment remarkably increased the sum of intersects (*p* = 0.0054; Figures 5L,M), ramification index (*p* = 0.0048; Figures 5L,N), and ending radius (*p* = 0.0456; Figures 5L,O) in the detailed Sholl analysis, and upregulated the percentage of A1-type astrocytes (*p* = 0.0013; Figures 5P,R), downregulated A2-type-astrocytes (*p* < 0.0001; Figures 5Q,S) in mice treated with ICH plus Dex, and GSK1016790A exposure compared to ICH plus Dex and vehicle (*versus* ICH + Dex + vehicle). In addition, there was no significant difference of average speed between mice exposed to ICH + Dex + vehicle and ICH + Dex + GSK groups (Figure 5G). These findings confirm that the TRPV4 signal is responsible for Dex-mediated neuroprotective effects post-ICH.

### Activation of the TPRV4 Signal in Astrocytes Eliminates Dex-Mediated Anti-Oxidative and Anti-Inflammatory Response *In Vitro*

**FIGURE 6:**
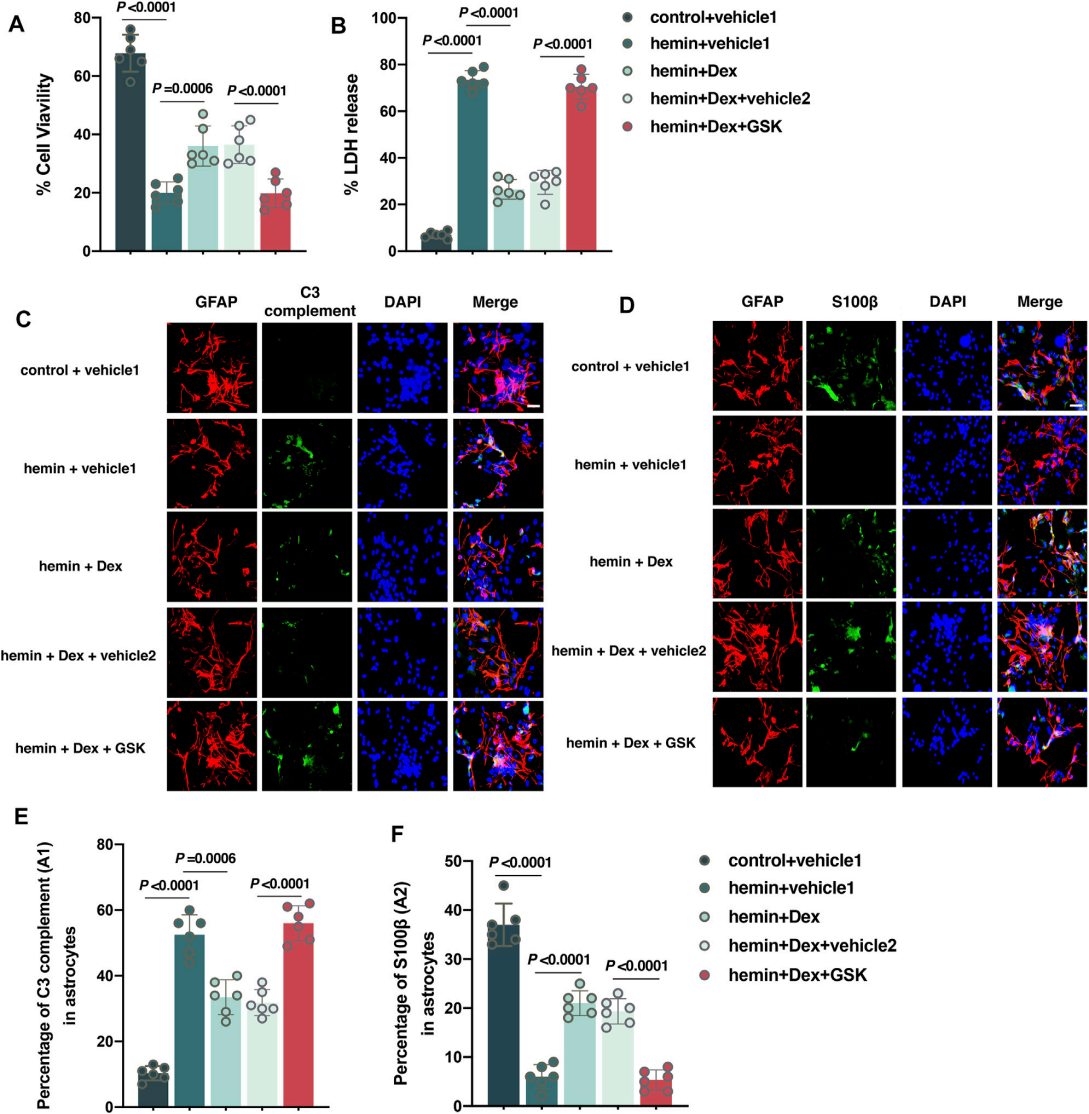
Activation of the TPRV4 signal in astrocytes eliminates dexmedetomidine-mediated neuroprotective effects *in vitro*. **(A,B)** Changes in the cell viability and LDH releases in cultured astrocytes caused by the indicated stimuli. **(C)** Representative photomicrographs of GFAP and C3 complement plus DAPI staining (GFAP, red; C3 complement, green; DAPI, blue). 400× magnification, scale bar = 20 μm. **(D)** Representative photomicrographs of GFAP and S100β plus DAPI staining (GFAP, red; S100β, green; DAPI, blue). 200× magnification, scale bar = 20 μm. **(E)** The percentage of GFAP combined with C3 complement-positive cells (A1-type) caused by the indicated stimuli. **(F)** The percentage of GFAP combined with S100β-positive cells (A2-type) caused by the indicated stimuli. Data are presented as the mean ± SD (*n* = 12 or 6). Hemin: 30 μM of hemin for 12 h was used to imitate ICH *in vitro*; Dex: Dex (1 μM) was administered into cell culture 30 min after hemin treatment; GSK: GSK1016790A (1 μM) was administered into cell culture 30 min after hemin treatment.

It was concluded that astrocytes under the ICH condition responded to Dex administration *in vivo*. Furthermore, cultured astrocytes exposed to hemin were used to establish an ICH model *in vitro*. The results of the CCK-8 assay showed that astrocyte viability of the hemin + vehicle 1 group was significantly lower than that of the control + vehicle 1 group (*versus* control + vehicle 1, *p* < 0.0001; Figure 6A). However, Dex significantly increased astrocyte viability in the hemin + Dex group compared to hemin + vehicle 1 group (*versus* hemin + vehicle 1, *p* = 0.0006; Figure 6A), whereas GSK1016790A reversed the increases of viability in the hemin + Dex + GSK group (*versus* hemin + Dex + vehicle 2, *p* < 0.0001; Figure 6A). In contrast, the data of LDH-releasing assay revealed that astrocyte injury dramatically elevated in the hemin + vehicle 1 group, compared to the control + vehicle 1 group (*versus* control + vehicle 1, *p* < 0.0001; Figure 6B). Dex resulted in a remarkable reduction in the astrocyte injury in the hemin + Dex group (*versus* hemin + vehicle 1, *p* < 0.0001; Figure 6B), whilst GSK1016790A abolished the astrocyte injury reduction mediated by Dex in the hemin + Dex + GSK group (*versus* hemin + Dex + vehicle 2, *p* < 0.0001; Figure 6B). Dex was found to produce antioxidative and anti-inflammatory responses against hemin-induced astrocytic injury. Astrocytes exposed to Dex treatment demonstrated a significant reduction in A1-type astrocytes indicated by C3 complement (*p* = 0.0006; Figures 6C,E), but a remarkable increase in A2-type astrocytes indicated by S100β (*p* < 0.0001; Figures 6D,F) (hemin + vehicle 1 *versus* hemin + Dex + vehicle 2), while GSK1016790A treatment led to astrocytes returning to a status similar to the hemin + vehicle 1 group (for A1, *p* < 0.0001; Figures 6C,E) (for A2, *p* < 0.0001; Figures 6D,F) (hemin + Dex + GSK *versus* hemin + Dex + vehicle 2). Taken together, these findings indicate that activation of the TPRV4 signal in astrocytes eliminates Dex-mediated antioxidative and anti-inflammatory response under hemin exposure *in vitro*.

## Discussion

This study showed that Dex treatment in mice with ICH exhibited improved anxiety-like behaviors, as indicated by an increased time and distance in the open arm, an upregulated time and distance in the central zone, an elevated total SOD content, a reduced MDA content, and a decreased number of A1-type astrocytes, a restored number of A2-type astrocytes, whereas an agonist of TRPV4 channels (GSK1016790A) partially reversed these changes in mice. Taken together, these results indicate that Dex treatment alleviated ICH-induced anxiety-behaviors via modulation of TRPV4 channels in the astrocyte.

In the present study, we established a mouse model of moderate ICH using an injection of 20 μl autologous arterial blood, which is indicated by the mNSS 7–12. Our current data suggested that the mNSS was increased 24 h post-ICH, which indicates neurologic deficits at early stage after ICH. Moreover, we also found that there was no significant difference of motor function as indicated by the mNSS 30 days post-ICH, which is consistent with a previous study (An et al., 2022). Growing evidence suggested that the neurologic deficit of mice exposed to mild and moderated ICH is gradually attenuated (Bonsack et al., 2016; Diaz Diaz et al., 2020), but severe ICH induced by 100 μl autologous arterial blood can result in a long-term neurologic deficit (MacLellan et al., 2008). Results revealed that an autologous blood injection model *in vivo* induced significant emotional changes such as anxiety-like behaviors, which is consistent with the results of previous publications (Li et al., 2017; Luo et al., 2019). Although multiple pathological mechanisms have been demonstrated in the pathophysiological process of ICH, increasing evidence suggest that the oxidative stress and inflammatory response in the striatum are the main causes toward the progression of ICH (Duan et al., 2016; Zhang et al., 2017). In this study, we chose MDA and SOD contents as typical examples of oxidative stress, which are utilized in numerous other studies (Koskenkorva-Frank et al., 2013; El Tabaa et al., 2017). We found that ICH exposure results in the aggravation of oxidative stress. Hayashi et al. (2020) reported oxidative stress as indicated by increased levels of nicotinamide adenine dinucleotide phosphate (NADPH) oxidase subunits, cyclooxygenases-2 (COX-2), and SOD was observed in the long-term after ICH. Inflammatory A1-type astrocytes, which release neurotoxic factors, induce neuronal injury and degeneration (Cunha, 2008). In addition, the ramification of astrocytes indicated by the Sholl analysis was significantly increased. In contrast, A2-type astrocytes are reported to take part in neuroprotection against ischemia/reperfusion injury, traumatic brain injury, and neurodegenerative disease (Fan and Huo, 2021). We also observed the transformation from A1-to A2-type astrocytes under the ICH condition. It has been reported that Dex exerts excellent cytoprotective effects against oxidative stress and inflammatory response in ischemia- and hemorrhage-associated diseases (Jiang et al., 2017; Liu H. et al., 2020). More specifically, Dex administration has been suggested to have a neuroprotective effect in mice by suppressing the ICH-induced inflammatory response (Song and Zhang, 2019). Consistent with previous publications (Hwang et al., 2013; Huang and Jiang, 2019), we found that Dex remarkably reduced ICH-induced oxidative stress and inflammatory response. Taken together, these results suggest that the neuroprotective effects of Dex may be associated with the inhibition of ICH-induced excessive oxidative stress and neurotoxic response.

TRPV4 channels are reported to act as chemical, mechanical stimuli, and osmotic sensors, and changes in the internal environment can induce the activation of TRPV4 (Liedtke and Kim, 2005; Suzuki et al., 2013). TRPV4 can sense mechanical stimulation, eicosanoid metabolites, and cell swelling, and then, it can adjust its state (Liu et al., 2021). Numerous publications have demonstrated that oxidative stress after ICH results in cell swelling and brain edema (Zhang Z. et al., 2018; Liu XC. et al., 2020). The pathophysiological process of the internal environment further induces the activation of the TRPV4-channel opening (Vriens et al., 2004). TRPV4 is mainly expressed in astrocytes, which are the predominant cell type in the brain, and perform key functions vital to the central nervous system’s physiology, including blood–brain barrier formation and maintenance, synaptogenesis, neurotransmission, and metabolic regulation (Kitchen et al., 2020). It was reported that TRPV4-mediated calcium influx in astrocytes contributes to neuronal activation, enhancing the accompanying vasodilation (Ye et al., 2018). Moreover, a previous study suggested that approximately 30% of astrocytes in the brain possess TRPV4, and TRPV4 activation in astrocytes results in the enhancement of synaptic activity through the release of excitatory glutamate (Shibasaki et al., 2014). Our data showed that the agonist of TRPV4 abolished the improvement of anxiety-like behaviors induced by ICH exposure. In addition, GSK1016790A not only inhibited astrocytic activation indicated by increased ramification and A1 phenotype in mice exposed to ICH plus Dex treatment, but also eliminated astrocytic activation after hemin exposure *in vivo*. These results indicated that the inhibition of TRPV4-channel opening in astrocytes may be related to the neuroprotective effects of Dex under the ICH condition.

In conclusion, Dex administration improves ICH-induced anxiety-like behaviors in mice, and the mechanism may be associated with attenuation of neuronal activation regulated by astrocytic transformation from A1-to A2-type *via* the inhibition of TRPV4-channel opening. Although further studies including downstream signals of TRPV4-channel are required, we suggest that Dex has the potential to provide therapy for ICH.

## Data Availability

The original contributions presented in the study are included in the article/Supplementary Material, further inquiries can be directed to the corresponding author.

## References

[B1] An P. Zhao X. C. Liu M. J. You Y. Q. Li J. Y. (2022). Gender-based Differences in Neuroprotective Effects of Hydrogen Gas against Intracerebral Hemorrhage-Induced Depression. Neurochem. Int. 153, 105276. 10.1016/j.neuint.2022.105276 34995727

[B2] Bao N. Dai D. (2020). Dexmedetomidine Protects against Ischemia and Reperfusion-Induced Kidney Injury in Rats. Mediators Inflamm. 2020, 2120971. 10.1155/2020/2120971 32317860 PMC7157761

[B3] Bonsack F. Alleyne C. H. Jr. Sukumari-Ramesh S. (2016). Augmented Expression of TSPO after Intracerebral Hemorrhage: a Role in Inflammation? J. Neuroinflammation 13, 151. 10.1186/s12974-016-0619-2 27315802 PMC4912814

[B4] Chen Y. Feng X. Hu X. Sha J. Li B. Zhang H. (20182018). Dexmedetomidine Ameliorates Acute Stress-Induced Kidney Injury by Attenuating Oxidative Stress and Apoptosis through Inhibition of the ROS/JNK Signaling Pathway. Oxid Med. Cell Longev 2018, 4035310. 10.1155/2018/4035310 PMC6140004 30250633

[B5] Cunha R. A. (2008). Different Cellular Sources and Different Roles of Adenosine: A1 Receptor-Mediated Inhibition through Astrocytic-Driven Volume Transmission and Synapse-Restricted A2A Receptor-Mediated Facilitation of Plasticity. Neurochem. Int. 52, 65–72. 10.1016/j.neuint.2007.06.026 17664029

[B6] Diaz Diaz A. C. Shearer J. A. Malone K. Waeber C. (2020). Acute Treatment with Fingolimod Does Not Confer Long-Term Benefit in a Mouse Model of Intracerebral Haemorrhage. Front. Pharmacol. 11, 613103. 10.3389/fphar.2020.613103 33488389 PMC7821021

[B7] Duan X. Wen Z. Shen H. Shen M. Chen G. (2016). Intracerebral Hemorrhage, Oxidative Stress, and Antioxidant Therapy. Oxid Med. Cell Longev 2016, 1203285. 10.1155/2016/1203285 27190572 PMC4848452

[B8] Dunn K. M. Hill-Eubanks D. C. Liedtke W. B. Nelson M. T. (2013). TRPV4 Channels Stimulate Ca2+-Induced Ca2+ Release in Astrocytic Endfeet and Amplify Neurovascular Coupling Responses. Proc. Natl. Acad. Sci. U S A. 110, 6157–6162. 10.1073/pnas.1216514110 23530219 PMC3625327

[B9] El Tabaa M. M. Sokkar S. S. Ramadan E. S. Abd El Salam I. Z. Zaid A. (2017). Neuroprotective Role of Ginkgo Biloba against Cognitive Deficits Associated with Bisphenol A Exposure: An Animal Model Study. Neurochem. Int. 108, 199–212. 10.1016/j.neuint.2017.03.019 28366721

[B10] Fan Y. Y. Huo J. (2021). A1/A2 Astrocytes in central Nervous System Injuries and Diseases: Angels or Devils? Neurochem. Int. 148, 105080. 10.1016/j.neuint.2021.105080 34048845

[B11] Filosa J. A. Yao X. Rath G. (2013). TRPV4 and the Regulation of Vascular Tone. J. Cardiovasc. Pharmacol. 61, 113–119. 10.1097/FJC.0b013e318279ba42 23107877 PMC3564998

[B12] Gu J. Chen J. Xia P. Tao G. Zhao H. Ma D. (2011). Dexmedetomidine Attenuates Remote Lung Injury Induced by Renal Ischemia-Reperfusion in Mice. Acta Anaesthesiol Scand. 55, 1272–1278. 10.1111/j.1399-6576.2011.02526.x 22092133

[B13] Hayashi K. Hasegawa Y. Takemoto Y. Cao C. Mukasa A. Kim-Mitsuyama S. (2020). Enhanced Oxidative Stress Contributes to Worse Prognosis and Delayed Neurofunctional Recovery after Striatal Intracerebral Hemorrhage in 5XFAD Mice. Eur. J. Neurosci. 51, 1806–1814. 10.1111/ejn.14596 31621130

[B14] Huang J. Jiang Q. (2019). Dexmedetomidine Protects against Neurological Dysfunction in a Mouse Intracerebral Hemorrhage Model by Inhibiting Mitochondrial Dysfunction-Derived Oxidative Stress. J. Stroke Cerebrovasc. Dis. 28, 1281–1289. 10.1016/j.jstrokecerebrovasdis.2019.01.016 30797643

[B15] Hwang L. Choi I. Y. Kim S. E. Ko I. G. Shin M. S. Kim C. J. (2013). Dexmedetomidine Ameliorates Intracerebral Hemorrhage-Induced Memory Impairment by Inhibiting Apoptosis and Enhancing Brain-Derived Neurotrophic Factor Expression in the Rat hippocampus. Int. J. Mol. Med. 31, 1047–1056. 10.3892/ijmm.2013.1301 23503673

[B16] Ji M. H. Jia M. Zhang M. Q. Liu W. X. Xie Z. C. Wang Z. Y. (2014). Dexmedetomidine Alleviates Anxiety-like Behaviors and Cognitive Impairments in a Rat Model of post-traumatic Stress Disorder. Prog. Neuropsychopharmacol. Biol. Psychiatry 54, 284–288. 10.1016/j.pnpbp.2014.06.013 25004167

[B17] Jiang L. Hu M. Lu Y. Cao Y. Chang Y. Dai Z. (2017). The Protective Effects of Dexmedetomidine on Ischemic Brain Injury: A Meta-Analysis. J. Clin. Anesth. 40, 25–32. 10.1016/j.jclinane.2017.04.003 28625441

[B18] Kaur J. Conti E. (2021). Dataset on Inflammation Induced after Lumbar Puncture. Data Brief 34, 106729. 10.1016/j.dib.2021.106729 33521177 PMC7820377

[B19] Keating G. M. (2015). Dexmedetomidine: A Review of its Use for Sedation in the Intensive Care Setting. Drugs 75, 1119–1130. 10.1007/s40265-015-0419-5 26063213

[B20] King A. Szekely B. Calapkulu E. Ali H. Rios F. Jones S. (2020). The Increased Densities, but Different Distributions, of Both C3 and S100A10 Immunopositive Astrocyte-like Cells in Alzheimer's Disease Brains Suggest Possible Roles for Both A1 and A2 Astrocytes in the Disease Pathogenesis. Brain Sci. 10, 503. 10.3390/brainsci10080503 PMC7463428 32751955

[B21] Kitchen P. Salman M. M. Halsey A. M. Clarke-Bland C. Macdonald J. A. Ishida H. (2020). Targeting Aquaporin-4 Subcellular Localization to Treat Central Nervous System Edema. Cell 181, 784–e19. e719. 10.1016/j.cell.2020.03.037 32413299 PMC7242911

[B22] Koivunen R. J. Harno H. Tatlisumak T. Putaala J. (2015). Depression, Anxiety, and Cognitive Functioning after Intracerebral Hemorrhage. Acta Neurol. Scand. 132, 179–184. 10.1111/ane.12367 25639837

[B23] Koskenkorva-Frank T. S. Weiss G. Koppenol W. H. Burckhardt S. (2013). The Complex Interplay of Iron Metabolism, Reactive Oxygen Species, and Reactive Nitrogen Species: Insights into the Potential of Various Iron Therapies to Induce Oxidative and Nitrosative Stress. Free Radic. Biol. Med. 65, 1174–1194. 10.1016/j.freeradbiomed.2013.09.001 24036104

[B24] Lee B. M. Jang Y. Park G. Kim K. Oh S. H. Shin T. J. (2020). Dexmedetomidine Modulates Transient Receptor Potential Vanilloid Subtype 1. Biochem. Biophys. Res. Commun. 522, 832–837. 10.1016/j.bbrc.2019.11.146 31796207

[B25] Li M. Ren H. Sheth K. N. Shi F. D. Liu Q. (2017). A TSPO Ligand Attenuates Brain Injury after Intracerebral Hemorrhage. Faseb j 31, 3278–3287. 10.1096/fj.201601377RR 28416580 PMC5503714

[B26] Liang H. Liu H. Z. Wang H. B. Zhong J. Y. Yang C. X. Zhang B. (2017). Dexmedetomidine Protects against Cisplatin-Induced Acute Kidney Injury in Mice through Regulating Apoptosis and Inflammation. Inflamm. Res. 66, 399–411. 10.1007/s00011-017-1023-9 28224201

[B27] Liedtke W. Kim C. (2005). Functionality of the TRPV Subfamily of TRP Ion Channels: Add Mechano-TRP and Osmo-TRP to the Lexicon!. Cell Mol Life Sci 62, 2985–3001. 10.1007/s00018-005-5181-5 16314934 PMC11139190

[B28] Liu H. Busl K. M. Doré S. (2020a). Role of Dexmedetomidine in Aneurysmal Subarachnoid Hemorrhage: A Comprehensive Scoping Review. J. Neurosurg. Anesthesiol 34, 176–182. 10.1097/ana.0000000000000728 33060552

[B29] Liu L. Guo M. Lv X. Wang Z. Yang J. Li Y. (2021). Role of Transient Receptor Potential Vanilloid 4 in Vascular Function. Front. Mol. Biosci. 8, 677661. 10.3389/fmolb.2021.677661 33981725 PMC8107436

[B30] Liu X. C. Wu C. Z. Hu X. F. Wang T. L. Jin X. P. Ke S. F. (2020b). Gastrodin Attenuates Neuronal Apoptosis and Neurological Deficits after Experimental Intracerebral Hemorrhage. J. Stroke Cerebrovasc. Dis. 29, 104483. 10.1016/j.jstrokecerebrovasdis.2019.104483 31727597

[B31] Liu Z. Chopp M. (2016). Astrocytes, Therapeutic Targets for Neuroprotection and Neurorestoration in Ischemic Stroke. Prog. Neurobiol. 144, 103–120. 10.1016/j.pneurobio.2015.09.008 26455456 PMC4826643

[B32] Luo Q. Li D. Bao B. Wan X. Pan B. Tu J. (2019). NEMO-binding Domain Peptides Alleviate Perihematomal Inflammation Injury after Experimental Intracerebral Hemorrhage. Neuroscience 409, 43–57. 10.1016/j.neuroscience.2019.04.041 31047976

[B33] Maclellan C. L. Silasi G. Poon C. C. Edmundson C. L. Buist R. Peeling J. (2008). Intracerebral Hemorrhage Models in Rat: Comparing Collagenase to Blood Infusion. J. Cereb. Blood Flow Metab. 28, 516–525. 10.1038/sj.jcbfm.9600548 17726491

[B34] Martin-Fernandez M. Jamison S. Robin L. M. Zhao Z. Martin E. D. Aguilar J. (2017). Synapse-specific Astrocyte Gating of Amygdala-Related Behavior. Nat. Neurosci. 20, 1540–1548. 10.1038/nn.4649 28945222 PMC5903286

[B35] Mei B. Li J. Zuo Z. (2021). Dexmedetomidine Attenuates Sepsis-Associated Inflammation and Encephalopathy via central α2A Adrenoceptor. Brain Behav. Immun. 91, 296–314. 10.1016/j.bbi.2020.10.008 33039659 PMC7749843

[B36] Murayama Y. Takao H. Ishibashi T. Saguchi T. Ebara M. Yuki I. (2016). Risk Analysis of Unruptured Intracranial Aneurysms: Prospective 10-Year Cohort Study. Stroke 47, 365–371. 10.1161/STROKEAHA.115.010698 26742803

[B37] Rombolà L. Tridico L. Scuteri D. Sakurada T. Sakurada S. Mizoguchi H. (2017). Bergamot Essential Oil Attenuates Anxiety-like Behaviour in Rats. Molecules 22, 614. 10.3390/molecules22040614 PMC6154596 28398260

[B38] Sallinen H. Sairanen T. Strbian D. (2019). Quality of Life and Depression 3 Months after Intracerebral Hemorrhage. Brain Behav. 9, e01270. 10.1002/brb3.1270 30907075 PMC6520301

[B39] Shen J. Tu L. Chen D. Tan T. Wang Y. Wang S. (2019). TRPV4 Channels Stimulate Ca2+-Induced Ca2+ Release in Mouse Neurons and Trigger Endoplasmic Reticulum Stress after Intracerebral Hemorrhage. Brain Res. Bull. 146, 143–152. 10.1016/j.brainresbull.2018.11.024 30508606

[B40] Shibasaki K. Ikenaka K. Tamalu F. Tominaga M. Ishizaki Y. (2014). A Novel Subtype of Astrocytes Expressing TRPV4 (Transient Receptor Potential Vanilloid 4) Regulates Neuronal Excitability via Release of Gliotransmitters. J. Biol. Chem. 289, 14470–14480. 10.1074/jbc.M114.557132 24737318 PMC4031503

[B41] Song H. L. Zhang S. B. (2019). Therapeutic Effect of Dexmedetomidine on Intracerebral Hemorrhage via Regulating NLRP3. Eur. Rev. Med. Pharmacol. Sci. 23, 2612–2619. 10.26355/eurrev_201903_17411 30964190

[B42] Suzuki T. Notomi T. Miyajima D. Mizoguchi F. Hayata T. Nakamoto T. (2013). Osteoblastic Differentiation Enhances Expression of TRPV4 that Is Required for Calcium Oscillation Induced by Mechanical Force. Bone 54, 172–178. 10.1016/j.bone.2013.01.001 23314072

[B43] Vriens J. Watanabe H. Janssens A. Droogmans G. Voets T. Nilius B. (2004). Cell Swelling, Heat, and Chemical Agonists Use Distinct Pathways for the Activation of the Cation Channel TRPV4. Proc. Natl. Acad. Sci. U S A. 101, 396–401. 10.1073/pnas.0303329101 14691263 PMC314196

[B44] Wang Z. Zhou F. Dou Y. Tian X. Liu C. Li H. (2018). Melatonin Alleviates Intracerebral Hemorrhage-Induced Secondary Brain Injury in Rats via Suppressing Apoptosis, Inflammation, Oxidative Stress, DNA Damage, and Mitochondria Injury. Transl Stroke Res. 9, 74–91. 10.1007/s12975-017-0559-x 28766251 PMC5750335

[B45] Wang Z. Zhou L. An D. Xu W. Wu C. Sha S. (2019). TRPV4-induced Inflammatory Response Is Involved in Neuronal Death in Pilocarpine Model of Temporal Lobe Epilepsy in Mice. Cell Death Dis 10, 386. 10.1038/s41419-019-1612-3 31097691 PMC6522539

[B46] Wu L. P. Kang W. Q. (2020). Effect of Dexmedetomidine for Sedation and Cognitive Function in Patients with Preoperative Anxiety Undergoing Carotid Artery Stenting. J. Int. Med. Res. 48, 300060520938959. 10.1177/0300060520938959 32972265 PMC7522831

[B47] Yang L. Wang M. Guo Y. Y. Sun T. Li Y. J. Yang Q. (2016). Systemic Inflammation Induces Anxiety Disorder through CXCL12/CXCR4 Pathway. Brain Behav. Immun. 56, 352–362. 10.1016/j.bbi.2016.03.001 26952745

[B48] Ye L. Xu M. Hu M. Zhang H. Tan X. Li Q. (2018). TRPV4 Is Involved in Irisin-Induced Endothelium-dependent Vasodilation. Biochem. Biophys. Res. Commun. 495, 41–45. 10.1016/j.bbrc.2017.10.160 29097199

[B49] Zhang Y. Jia S. Gao T. Zhang R. Liu Z. Wang Y. (2018a). Dexmedetomidine Mitigate Acute Lung Injury by Inhibiting IL-17-induced Inflammatory Reaction. Immunobiology 223, 32–37. 10.1016/j.imbio.2017.10.017 29030006

[B50] Zhang Z. Wu Y. Yuan S. Zhang P. Zhang J. Li H. (2018b). Glutathione Peroxidase 4 Participates in Secondary Brain Injury through Mediating Ferroptosis in a Rat Model of Intracerebral Hemorrhage. Brain Res. 1701, 112–125. 10.1016/j.brainres.2018.09.012 30205109

[B51] Zhang Z. Zhang Z. Lu H. Yang Q. Wu H. Wang J. (2017). Microglial Polarization and Inflammatory Mediators after Intracerebral Hemorrhage. Mol. Neurobiol. 54, 1874–1886. 10.1007/s12035-016-9785-6 26894396 PMC4991954

[B52] Zhu H. Wang Z. Yu J. Yang X. He F. Liu Z. (2019). Role and Mechanisms of Cytokines in the Secondary Brain Injury after Intracerebral Hemorrhage. Prog. Neurobiol. 178, 101610. 10.1016/j.pneurobio.2019.03.003 30923023

